# Detection of *Leishmania infantum* DNA in Sand Flies and Assessment of Zoonotic Transmission Risk in Central Honduras: A One Health Approach

**DOI:** 10.3390/tropicalmed11080219

**Published:** 2026-08-05

**Authors:** Elisa Alcántara-Henríquez, Hugo O. Valdivia, Gissella M. Vásquez, Joel García, Estela Guevara, Juan F. Sanchez, Adalid Palma, Arnold Houghton, Allan Iván Izaguirre González, Marcia D. Laurenti, Wilfredo Sosa-Ochoa

**Affiliations:** 1 Microbiology Research Institute, Facultad de Ciencias, Universidad Nacional Autónoma de Honduras, Tegucigalpa 11101, Honduras; elisa.alcantara@unah.edu.hn (E.A.-H.); joel.garcia@unah.edu.hn (J.G.); 2 Department of Parasitology, U.S. Naval Medical Research Unit SOUTH (NAMRU SOUTH), Bellavista 07006, Peru; hugo.o.valdivia.ln@health.mil (H.O.V.); juan.sanchez209.ln@health.mil (J.F.S.); 3 Department of Entomology, U.S. Naval Medical Research Unit SOUTH (NAMRU SOUTH), Bellavista 07006, Peru; gissella.m.vasquez.ln@health.mil (G.M.V.); adalid.palma@culmen.com (A.P.); 4 Maestría de Enfermedades Infecciosas y Zoonóticas, Facultad de Ciencias, Universidad Nacional Autónoma de Honduras, Tegucigalpa 11101, Honduras; zoila.guevara@unah.hn; 5 Culmen International, Alexandria, VA 22314, USA; 6 Departamento de Químico, Biológico y Salud, Universidad Nacional Autónoma de Honduras, Campus Comayagua, Comayagua 12101, Honduras; arnold.houghton@unah.edu.hn; 7 Facultad de Posgrados, Universidad Tecnológica Centroamericana, Tegucigalpa 11101, Honduras; allanizaguirre@unitec.edu; 8 Laboratory of Pathology of Infectious Diseases (LIM50), Department of Pathology, Medical School, São Paulo University, São Paulo 01246-903, SP, Brazil; mdlauren@usp.br

**Keywords:** *Leishmania infantum*, non-ulcerated leishmaniasis, sand flies

## Abstract

Integrated One Health studies are essential for assessing the risks of leishmaniasis transmission. In Honduras, the most prevalent clinical presentation of leishmaniasis is the non-ulcerated cutaneous form caused by *Leishmania infantum*; however, research on its transmission dynamics remains limited. This study aimed to characterize the eco-epidemiology of leishmaniasis and identify potential zoonotic risk factors in the municipality of Ojos de Agua, Department of Comayagua, central Honduras, using a One Health approach. We collected sand flies using CDC light traps placed in intra- and peridomestic settings of households with a history of leishmaniasis. Concurrently, we evaluated domestic dogs and collected blood samples for serological and molecular testing. We also conducted active surveillance among community residents to identify human infections. We collected a total of 644 phlebotomine sand flies, including 186 females. We identified eight sandfly species, with *Lutzomyia* (*Lutzomyia*) *longipalpis* as the predominant species, followed by *Pintomyia* (*Pifanomyia*) *evansi*. Among the 64 engorged females screened, the overall PCR positivity rate for *Leishmania* spp. was 14.1% (9/64). Blood meal analysis of PCR-positive sand flies identified humans and pigs as blood and meal sources. Additionally, 18.6% (8/43) of domestic dogs were seropositive for *Leishmania* spp., and 23.3% (10/43) tested positive by PCR. DNA sequencing identified the infecting species as *Leishmania infantum*. We detected no active human cases during the study period. These findings highlight the value of a One Health approach for improving transmission risk monitoring in newly emerging areas with a history of ulcerative cutaneous leishmaniasis in Honduras.

## 1. Introduction

Leishmaniasis is a vector-borne parasitic disease caused by more than 20 species of the genus *Leishmania* (Kinetoplastida: Trypanosomatidae). The disease is transmitted through the bite of infective, hematophagous female sand flies [1]. It is widely distributed across tropical and subtropical regions of 99 countries in Europe, Africa, Asia, and the Americas [2], with transmission occurring predominantly in rural settings and among adult males [3,4].

In the Americas, leishmaniasis remains a major public health problem, particularly in rural, forested, and peri-urban areas. Because the disease is endemic in regions frequently used for military training and operations, it poses a persistent threat to military readiness and force health protection. For example, an outbreak of cutaneous leishmaniasis among Peruvian military personnel who trained in the Amazon Basin resulted in an attack rate of 25%, highlighting the high risk of infection associated with military activities in endemic areas [5].

Honduras, a Central American country, is endemic for four distinct clinical forms of leishmaniasis: cutaneous leishmaniasis (CL), mucosal leishmaniasis (ML), visceral leishmaniasis (VL), and non-ulcerated cutaneous leishmaniasis (NUCL). NUCL is an atypical variant of CL characterized by non-ulcerated papules or nodules, low parasite burdens, and a distinct cell-mediated immune profile, in contrast to the ulcerative lesions typical of classic CL [6,7,8,9,10]. The epidemiological burden of leishmaniasis in Honduras is predominantly cutaneous, with 5360 reported cases of CL and ML between 2020 and 2023, whereas only one case of VL was reported during the same period [11]. Furthermore, these clinical forms exhibit distinct geographic distributions and are associated with different *Leishmania* species and sandfly vectors.

The semi-arid regions of southern Honduras are endemic for NUCL and VL, both caused by *Leishmania infantum*. In contrast, the more humid northern region is characterized by a higher prevalence of CL and ML caused by parasites of the subgenus *Viannia* (*Leishmania braziliensis* and *Leishmania panamensis*) [4,12]. To date, 29 sandfly species have been reported in Honduras, including *Lutzomyia* (*Lutzomyia*) *longipalpis* and *Pintomyia* (*Pifanomyia*) *evansi*, both of which have been identified as potential vectors of NUCL and VL in the Mesoamerican Pacific region [6,10,13,14]. Dogs are widely considered the primary domestic reservoir of *Leishmania infantum*; however, evidence regarding their role in transmission remains inconsistent [15]. Some studies suggest that asymptomatic dogs either fail to infect sandfly vectors or do so at rates comparable to those of oligosymptomatic dogs. In contrast, other studies have demonstrated that asymptomatic dogs efficiently transmit *Leishmania infantum* to its primary vector, *Lutzomyia* (*Lutzomyia*) *longipalpis* [16]. A study conducted in southern Honduras reported that dogs may not represent an important source of vector infection in NUCL-endemic areas because parasites were absent from the skin, and parasite loads detected in the buffy coat were low [17].

To address these knowledge gaps, this study aimed to characterize the eco-epidemiology of leishmaniasis in the municipality of Ojos de Agua during August 2023 using a One Health approach. Specifically, we sought to identify potential vector species and characterize *Leishmania* species detected in sand flies and dogs. The findings may help inform prevention and control strategies for leishmaniasis in central Honduras and other endemic areas, thereby contributing to public health and force health protection in the region.

## 2. Materials and Methods

### 2.1. Study Site

**Figure 1:**
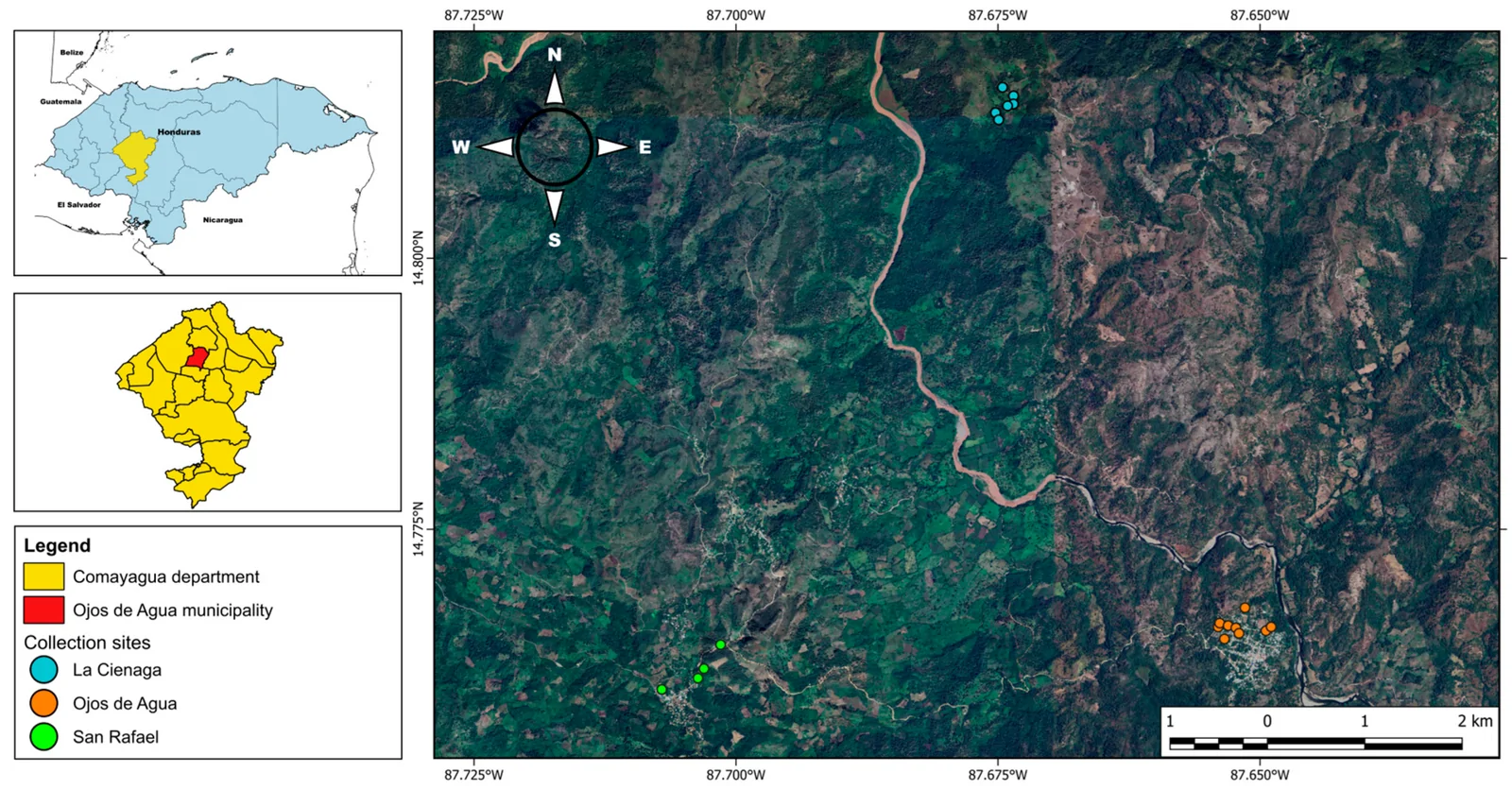
Collection sites in the municipality of Ojos de Agua, Department of Comayagua, central Honduras. The map was generated using the free and open-source software QGIS version 3.34.7 (https://qgis.org/).

The study was conducted in a rural community within the municipality of Ojos de Agua (14°45′49″ N, 87°39′01″ W), located in the Department of Comayagua in central Honduras. The municipality covers an area of 166.7 km^2^ (Figure 1) and comprises 28 villages with an estimated population of 11,006 inhabitants [18]. The region is characterized by subtropical dry forest, with temperatures ranging from 19 °C to 33 °C and an average annual rainfall of 3000 mm. Before this study, no baseline data were available on the etiological agents of leishmaniasis or the circulating phlebotomine sandfly species in the area. The terrain is predominantly mountainous and rugged, with a mean elevation of 1167 m above sea level (m.a.s.l.).

### 2.2. Study Design

We conducted a cross-sectional descriptive study in August 2023 in three rural villages (La Ciénaga, San Rafael, and Ojos de Agua) within the municipality of Ojos de Agua, Department of Comayagua. We selected nineteen households based on records from the Honduran Ministry of Health (MoH) documenting current or previous cases of CL. Concurrently, we conducted active surveillance through visual clinical screening for active skin lesions among students in local schools across the three villages. We clinically evaluated 102 human residents from the selected households. We sampled sand flies over five consecutive nights in August 2023 across the three villages. Collections were conducted from 6:00 p.m. to 6:00 a.m. using CDC miniature light traps (Model 512; John W. Hock, Gainesville, FL, USA). Two traps were installed at each household: one intradomiciliary and one peridomiciliary. Peridomiciliary traps were placed near domestic animal resting sites or adjacent to latrines. In addition, two extradomiciliary traps were installed in La Ciénaga in areas with minimal or no anthropogenic disturbance (e.g., crop fields and mountain trails), each located approximately 100 m from the peridomicile. We separated the collected specimens and stored them at −20 °C until processing. A total of 43 mongrel dogs of varying ages and sexes from the selected households were clinically evaluated for skin lesions and other signs of leishmaniasis. We collected 4 mL of whole blood from each dog by venipuncture into EDTA tubes. After centrifugation, plasma was separated and stored at −20 °C until analysis, whereas the buffy coat (BC) was stored at −70 °C for DNA extraction and PCR analysis. All biological samples were stored at the Centro de Investigaciones Genéticas (CIG), Universidad Nacional Autónoma de Honduras.

### 2.3. Serological Diagnosis in Dogs

Plasma samples were initially screened using an immunochromatographic assay based on dual-path platform (DPP) technology (DPP^®^ CVL rapid test, BioManguinhos, Rio de Janeiro, Brazil) according to the manufacturer’s instructions [19]. Subsequently, the samples were analyzed using an in-house enzyme-linked immunosorbent assay (ELISA) described by Laurenti et al. [20]. Briefly, polystyrene plates were coated with 100 μL of crude *Leishmania infantum* (MHOM/HN/2017/AMA-73) antigen at a concentration of 10 mg/mL in 0.1 M carbonate–bicarbonate buffer (pH 9.5). The plates were incubated overnight at 4 °C and then washed three times with 0.15 M phosphate-buffered saline (pH 7.2) containing 0.05% Tween-20 (PBS-T). Nonspecific binding sites were blocked with 100 μL of 10% powdered milk in PBS and incubated for 2 h at 37 °C in a humid chamber. After washing, duplicate test sera and controls diluted 1:400 in PBS-T were added (100 µL/well) and incubated at 37 °C for 1 h. The plates were then washed again, and alkaline phosphatase-conjugated anti-canine IgG (A40-123AP; Bethyl, Montgomery, TX, USA), diluted 1:2000 in PBS-T, was added and incubated at 37 °C for 45 min in a humid chamber. The color reaction was developed using a chromogenic substrate (1.0 mg/mL pNPP; Sigma, Virginia Beach, VA, USA) in 0.1 M carbonate–bicarbonate buffer (pH 9.5), followed by incubation at room temperature for 30 min. The reaction was stopped with 3 M NaOH, and absorbance was measured at 405 nm. The cutoff value was calculated as the mean absorbance plus three standard deviations.

### 2.4. Sandfly Taxonomic Identification

Phlebotomine sand flies were taxonomically identified based on diagnostic morphological characteristics. Specimens were prepared and mounted following the procedures described by Mejía et al. [6], and genus- and species-level identification was carried out using the keys developed by Young and Duncan [14] and Galati [21].

### 2.5. Data Analysis

The proportional abundance of sandfly species at each study site was calculated using the formula Pi = (*ni*/*N*) × 100, where *ni* represents the number of individuals of a given species, and *N* represents the total number of sand flies collected at each locality [22].

Species diversity was evaluated using the Shannon–Wiener diversity index (H′), calculated as *H′* = −∑[(*pi*) × *ln*(*pi*)], where *pi* represents the proportion of individuals belonging to species *i* [23]. This index was used to estimate species diversity at each study site. Species richness was defined as the total number of species recorded at each locality.

### 2.6. Dog and Sandfly DNA Extraction

DNA extraction from dog samples was performed using the Wizard^®^ Genomic DNA Purification Kit (Promega Corporation, Madison, WI, USA) according to the manufacturer’s protocol, and the extracted DNA was stored at −20 °C until use. The gut tissues of individual female and sand flies were dissected and placed in vials containing 60 μL of 5% Chelex^®^ 100 (Bio-Rad Laboratories, Hercules, CA, USA). The tissues were macerated, vortexed for 20 s, briefly centrifuged, and incubated at 97 °C for 30 min. A second centrifugation was then performed at 13,000 rpm for 10 min, after which the supernatant was transferred to sterile vials and stored at −20 °C. As an internal control for DNA extraction, the *cacophony* IVS6 gene present in the sandfly genome was amplified [24].

### 2.7. Leishmania Identification in Clinical Samples and Sand Flies

For the detection of the genus *Leishmania*, primers targeting a small fragment of kinetoplast DNA (kDNA) were used in a conventional endpoint PCR assay. The primer pair Leish1 (5′-AACTTTTCTCTGGTCCTCCGGGTAG-3′) and Leish2 (5′-ACCCCCAGTTTCCCGCC-3′) amplifies an approximately 120 bp product [25]. Amplification was performed using a commercial kit (Master Mix 2X; Promega). Each reaction contained 4 µL of target DNA and 0.6 µmol/L of each primer in a final volume of 20 µL. PCR cycling conditions consisted of an initial denaturation cycle at 94 °C for 5 min, followed by 35 cycles of denaturation at 95 °C for 15 s, annealing at 60 °C for 20 s, and extension at 72 °C for 60 s, with a final extension cycle at 72 °C for 10 min.

To characterize *Leishmania* species, ITS1 amplicons were directly sequenced. Amplification was performed using the primers LITSR (5′-CTGGATCATTTTCCGATG-3′) and L5.8S (5′-TGATACCACTTATCGCACTT-3′), which amplify a 300–350 bp product [26,27]. PCR amplification was performed using a commercial kit (Master Mix 2X; Promega). Each reaction contained 4 µL of target DNA and 0.6 µmol/L of each primer in a final volume of 20 µL. PCR cycling conditions consisted of an initial denaturation at 94 °C for 2 min, followed by 35 cycles of denaturation at 95 °C for 20 s, annealing at 60 °C for 30 s, and extension at 72 °C for 60 s, with a final extension at 72 °C for 6 min.

### 2.8. Identification of Sandfly Blood Meal Sources

Engorged female sand flies were screened by PCR amplification of a 215 bp fragment of the 12S ribosomal RNA gene using the primers L1085 (5′-CCCAAACTGGGATTAGATACCC-3′) and H1259 (5′-GTTTGCTGAAGATGGCGGTA-3′) [28,29,30]. Amplification was performed using a commercial kit (Master Mix 2X; Promega). Each reaction contained 4 µL of target DNA and 0.5 µmol/L of each primer in a final volume of 20 µL. PCR cycling conditions consisted of an initial denaturation at 95 °C for 5 min, followed by 35 cycles of denaturation at 95 °C for 30 s, annealing at 57 °C for 15 s, and extension at 72 °C for 30 s, with a final extension at 72 °C for 10 min.

### 2.9. Sequence Analysis

Amplicons were visualized on 2% ethidium bromide-stained agarose gels, purified, and sequenced by Psomagen Inc. (Rockville, MD, USA). The resulting sequences were aligned to generate consensus sequences using Geneious Prime 2024.0.5. For *Leishmania* identification, ITS1 consensus sequences were analyzed against the NCIB nonredundant (nr) GenBank database, and only sequences showing >98% similarity were retained. *Leishmania* DNA sequences obtained from clinical specimens and sand flies were deposited in the GenBank database under accession numbers PX619521, PX619522, PX619523, and PX619524.

To identify blood meal sources in sand flies, 12S rRNA sequences were analyzed using Geneious Prime version 2024.0.07 against the NCBI nonredundant (nr) GenBank database. Sequence similarity greater than 96% was considered indicative of species- or genus-level identification. The resulting 12S rRNA sequences were subsequently aligned using ClustalW with default parameters. The multiple sequence alignment was then used for phylogenetic reconstruction using the neighbor-joining method based on pairwise genetic distances and supported by 1000 bootstrap replicates, as implemented in MEGA version 12.0.14.

## 3. Results

### 3.1. Clinical Data in Dogs

A total of 43 dogs were evaluated in this study, including 20 males (46.5%) and 23 females (53.5%). The animals’ ages ranged from 2 months to 6 years, with 84% aged 3 years or less. Most animals appeared clinically healthy, with only a few exhibiting clinical signs suggestive of canine leishmaniasis, including onychogryphosis (4.6%, 2/43), skin lesions (6.9%, 3/43), and alopecia (18.6%, 8/43). Additionally, weight loss was observed in 70% (30/43) of the dogs evaluated (Table 1).

### 3.2. Serological and Parasitological Diagnosis in Dogs

Serological tests showed that 7.0% (3/43) of the dogs were positive by the DPP^®^ test, whereas 11.6% (5/43) were positive by ELISA. None of the DPP-positive dogs were ELISA-positive, indicating a lack of agreement between the two assays (*p* > 0.05). Parasitological diagnosis by polymerase chain reaction of the buffer coat (PCR-BC) identified *Leishmania* DNA in 23.2% (10/43) of the dogs. Of these PCR-positive dogs, only two exhibited clinical signs consistent with canine leishmaniasis (Table 2), whereas the remaining positive animals showed no apparent clinical manifestations at the time of evaluation.

### 3.3. Sandfly Diversity and Distribution

A total of 644 sand flies were collected, comprising 186 females (28.8%) and 458 males (71.2%). Eight species were identified, with *Lutzomyia* (*Lutzomyia*) *longipalpis*, *Pintomyia* (*Pifanomyia*) *evansi*, and *Dampfomyia* (*Coromyia*) *zeledoni*/*deleoni* being the most abundant species (Table 3). Based on trap location, the peridomiciliary ecotope yielded the highest proportion of sand flies (55.12%), followed by the intra-domiciliary (44.41%) and extradomiciliary ecotopes (0.47%).

**Figure 2:**
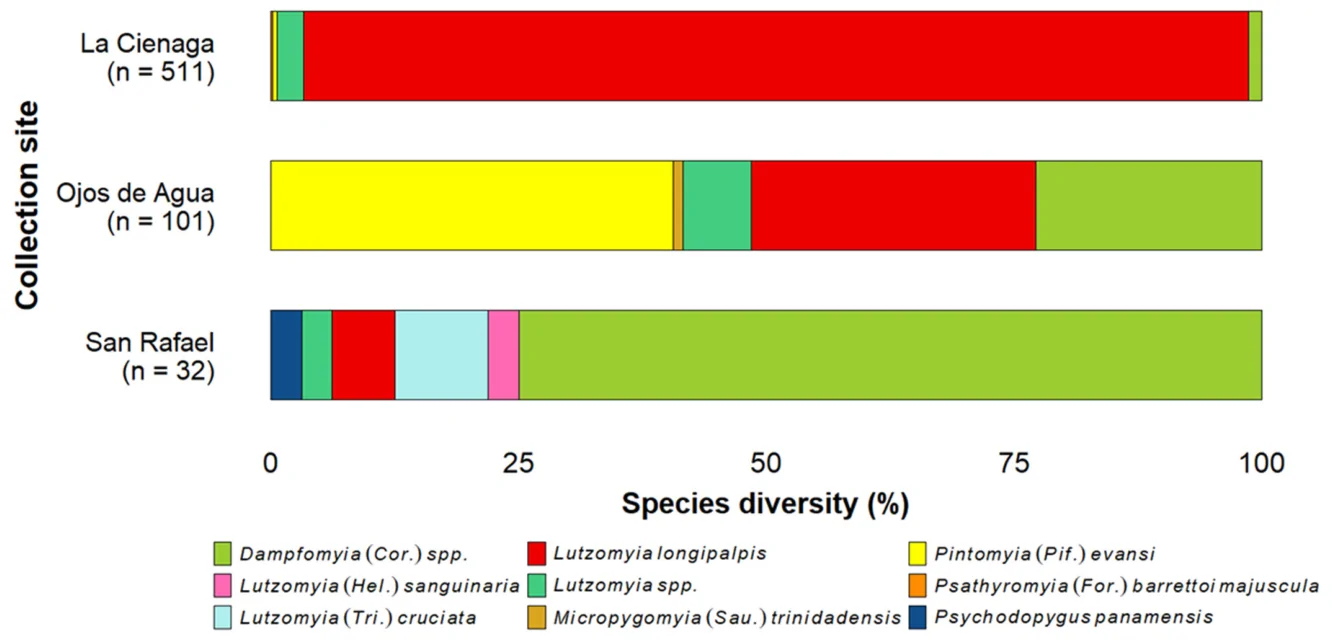
Comparison of sandfly species diversity among the three study sites.

Although the analysis of sandfly diversity revealed that La Ciénaga had the highest number of sand flies collected, with 511 individuals (79.3% of the total), Ojos de Agua accounted for 15.7% of all specimens and 40.5% of all *Pi. evansi* individuals. In La Ciénaga, *Lu. longipalpis* was the most abundant species, accounting for 95.3% of the specimens collected, followed by *Pi. evansi* (0.4%). In San Rafael, *Dampfomyia zeledoni*/*deleoni* was the predominant species (75.0%), followed by *Lu. cruciata* (9.4%) and *Lu. longipalpis* (6.3%). In Ojos de Agua, the most abundant species were *Pi. evansi* (40.6%), *Lu. longipalpis* (28.7%), and *Dampfomyia zeledoni*/*deleoni* (22.8%) (Figure 2). The Shannon diversity index indicated that Ojos de Agua had the highest species diversity (H’ = 1.2), whereas La Ciénaga exhibited lower diversity (H’ = 0.2).

### 3.4. Sandfly Blood Meal Sources

**Figure 3:**
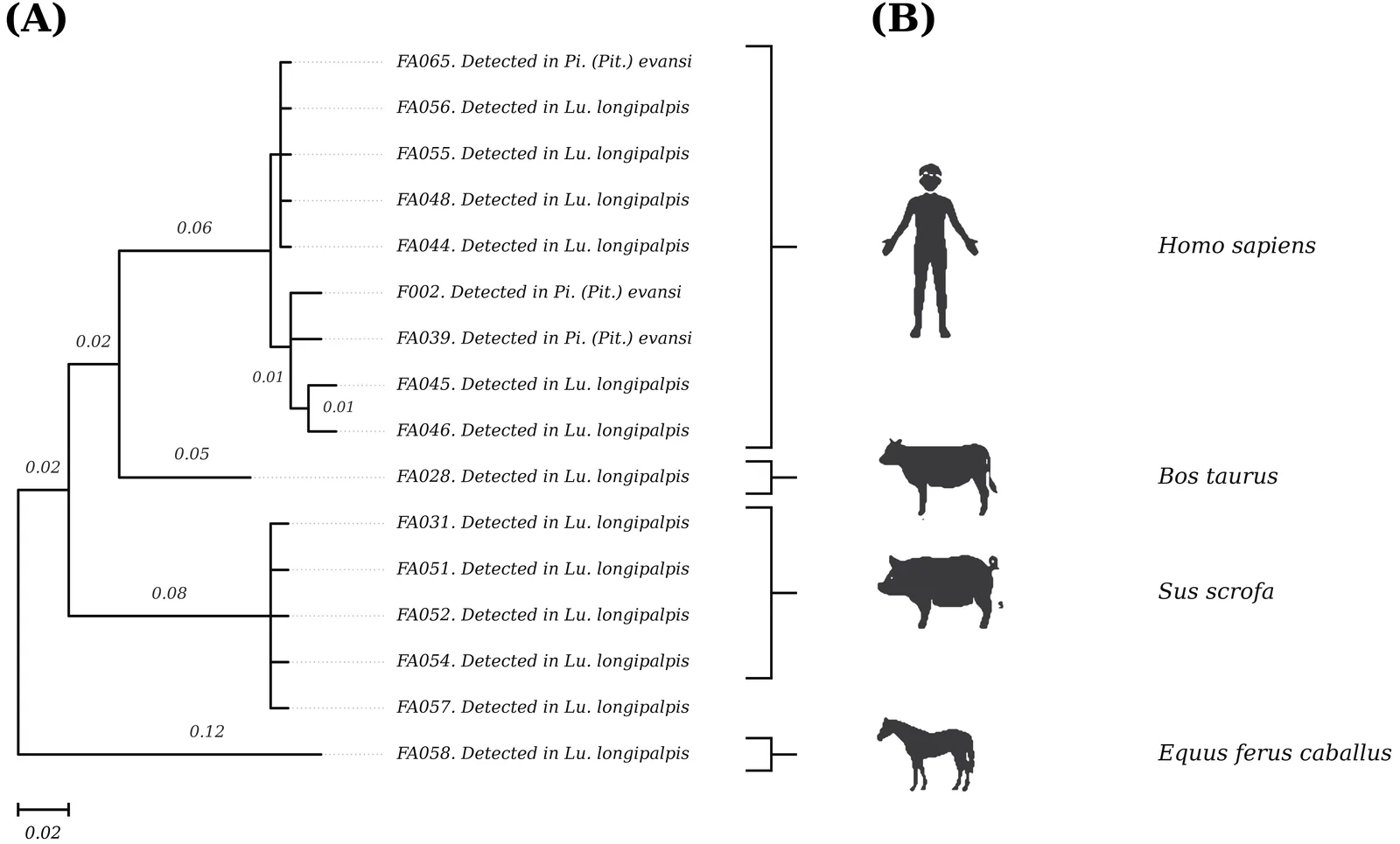
Neighbor-joining (NJ) phylogenetic tree and blood meal sources identified in sand flies collected in Ojos de Agua, Department of Comayagua, Honduras, using the 12S rRNA molecular marker. (**A**) Neighbor-joining dendrogram. (**B**) Identified vertebrate species.

Blood meals were detected in three sandfly species, and blood meal sources were successfully identified for *Lutzomyia* (*Lutzomyia*) *longipalpis* and *Pintomyia* (*Pifanomyia*) *evansi* (Table 4). No mixed-blood meals were detected. *Lu. longipalpis* exhibited both anthropophilic and zoophilic feeding behavior, with blood meals originating from humans (*Homo sapiens*) and domestic animals, including pigs (*Sus scrofa*), horses (*Equus caballus*), and cattle (*Bos taurus*). In contrast, all *Pi. evansi* specimens analyzed contained only human blood meals, suggesting anthropophilic feeding behavior in this population (Figure 3). No dog DNA was detected in any of the engorged female sand flies analyzed. The blood meal source could not be determined for *Dampfomyia* (*Coromyia*) *zeledoni*/*deleoni*.

### 3.5. Leishmania infantum DNA Detection in Dogs and Sand Flies

PCR screening of 64 sandfly specimens detected *Leishmania infantum* DNA in six *Lu.* (*Lu.*) *longipalpis* and three *Pi.* (*Pif.*) *evansi specimens*. The species-specific positivity rates were 12.2% (6/49) for *Lu.* (*Lu.*) *longipalpis* and 42.8% (3/7) for *Pi.* (*Pif.*) *evansi*, resulting in an overall positivity rate of 14.1% (9/64). All analyzed specimens successfully amplified a 220 bp fragment corresponding to the constitutive *cacophony* gene of *Lutzomyia* spp., confirming DNA integrity and the absence of PCR inhibitors.

In La Ciénaga, *Leishmania infantum* DNA was detected in five *Lu.* (*Lu.*) *longipalpis* specimens. Blood meal analysis identified human blood in two specimens and pig blood in three specimens. In San Rafael, *L. infantum* DNA was detected in one *Lu.* (*Lu.*) *longipalpis* specimen, which had fed exclusively on human blood. In Ojos de Agua, *Leishmania infantum* DNA was detected in three *Pi.* (*Pif.*) *evansi* specimens, all of which had also fed exclusively on human blood.

## 4. Discussion

The complex interactions among humans, sand flies, domestic animals, and wild reservoirs contribute to the endemic circulation of *Leishmania* spp. Therefore, implementing effective control, surveillance, and prevention strategies against leishmaniasis requires studies based on an integrated One Health approach, particularly in regions with a high risk of transmission [31]. Our study provides the first insights into sandfly species diversity and abundance, seroprevalence among potential canine reservoirs, and circulating *Leishmania* species in the region, establishing a baseline for understanding leishmaniasis transmission dynamics in Ojos de Agua, Honduras.

The findings of this study highlight the need for integrated surveillance systems and preventive strategies against sandfly-borne diseases in endemic regions. In Latin America, leishmaniasis poses an occupational risk for military personnel deployed in rural and forested environments where exposure to sandfly vectors is frequent; however, similar risks exist among communities residing in endemic areas. Effective prevention strategies include entomological surveillance, early case detection, personal protective measures such as insecticide-treated uniforms and repellents, environmental management, and health education. Therefore, integrated vector surveillance and control are essential components of public health strategies aimed at reducing *Leishmania* transmission among both high-risk groups and endemic populations [5,32].

In Honduras, efforts to characterize sandfly diversity and *Leishmania* species distribution have primarily focused on the southern region, which is endemic for NUCL and VL [6,10,17]. However, comparable information from the central region has remained limited. Our study was conducted in an area where CL has historically been the most frequently reported clinical form. According to records from the Comayagua Regional Department, 10 human CL cases were reported in the municipality of Ojos de Agua during 2022 and 2023 based on microscopic examination of skin scrapings [33]. However, we did not identify any individuals with lesions suspicious of CL, NUCL, or VL during our active surveillance. This represents an important limitation because the absence of human cases prevented direct assessment of the current transmission cycle. Therefore, interpretation regarding potential changes in transmission patterns should be made cautiously, as they are primarily based on indirect entomological and ecological evidence. The observed pattern may reflect changes in transmission dynamics or the influence of climatic and anthropogenic factors that reduce human–vector contact or alter the involvement of reservoir hosts, as reported in other regions of the Americas. Continuous epidemiological surveillance remains essential for improving our understanding of local transmission dynamics [34,35,36].

In the Americas, dogs are considered important domestic reservoirs of *Leishmania infantum* in areas where human VL occurs. Canine infections frequently precede human cases in zoonotic transmission cycles [15]. Although canine leishmaniasis has not been widely documented in central Honduras, our findings provide molecular evidence of parasite circulation among dogs in the study area, with 23% of evaluated animals testing positive by PCR. This finding suggests that canine infection may occur even in areas where canine leishmaniasis has not previously been recognized as endemic.

During the early stages of infection, parasites may be lysed by the host’s innate immune response. The resulting parasite remnants, which remain detectable by molecular methods, stimulate the adaptive immune response and promote the production of memory cells and specific antibodies. Consistent with this process, the overall seroprevalence of *Leishmania* infection in dogs was 18.6% (8/43), as determined by serological testing (DPP^®^ and/or ELISA), whereas parasite DNA was detected in 23.2% of the animals. This seroprevalence is comparable to that reported in other NUCL-endemic areas [7,17]. A notable finding was the lack of concordance between the two serological assays (DPP^®^ and ELISA). Because no dog tested positive by both assays, the combined seroprevalence was calculated as the total number of animals testing positive by either assay (3 DPP-positive + 5 ELISA-positive = 8), resulting in an overall seroprevalence of 18.6% (8/43). This discordance complicates the interpretation of the seroprevalence estimate and may reflect differences in assay performance, including variations in antigen composition and analytical sensitivity. The DPP assay used in this study is based on the recombinant antigen rK28, a chimeric protein comprising the K9, K26, and K39 antigens, whereas the ELISA employed crude *Leishmania infantum* antigen (MHOM/HN/2017/AMA-73), which may influence antibody detection. In addition, differences in the stage of the immune response detected by each assay may have contributed to the observed discordance in this Honduran epidemiological setting.

Depending on their genetic and immunological background, as well as nutritional status, most dogs infected with *Leishmania infantum* remain asymptomatic for extended periods [17]. Onychogryphosis, alopecia, weight loss, skin lesions, and lymphadenomegaly are among the principal clinical manifestations of canine leishmaniasis, which may progress to cachexia and death [37]. Nevertheless, infected dogs may remain asymptomatic or exhibit only subclinical signs, limiting the diagnostic value of clinical examination alone. The dogs evaluated in central Honduras generally did not exhibit clinical signs suggestive of canine leishmaniasis. However, previous serological studies have reported prevalence rates of 16% [7] and 60% [17], supporting the role of dogs in the local transmission cycle. In this present study, clinical manifestations were generally mild, with weight loss observed in 70% of the animals. However, this finding may not be clinically meaningful given the socioeconomic conditions of dog owners in the study area. Alopecia was observed in 18.6% of dogs, whereas onychogryphosis and skin lesions were present in 4.6% and 6.9% of animals, respectively. Most dogs exhibited only one or two clinical signs, which is insufficient to classify them as clinically symptomatic according to established criteria [38]. The weak association between clinical manifestations and infection observed in this study underscores the diagnostic challenges of canine leishmaniasis. Although 23.2% of the dogs were PCR-positive for *Leishmania infantum*, only two infected animals exhibited clinical signs compatible with the disease. These findings suggest that most infected dogs were asymptomatic or subclinically infected at the time of sampling, potentially contributing to the silent maintenance of the parasite within the canine population. Consequently, surveillance strategies based solely on clinical examination are likely to underestimate the true prevalence of infection.

Few studies have investigated the diversity of *Lutzomyia* species in Honduras. A study conducted in the 1990s on Isla del Tigre (Municipality of Amapala, Department of Valle) identified *Lu. longipalpis* as a vector of *Leishmania infantum* and described its ecological behavior [7,39]. Subsequently, Mejia et al. [6] expanded knowledge of *Lutzomyia* species diversity and provided important information on vector host preferences in southern Honduras. In the present study, *Lu.* (*Lu.*) *longipalpis* was the predominant sandfly species across all study sites (n = 502), followed by *Dampfomyia* (*Coromyia*) *zeledoni*/*deleoni* (n = 53) and *Pi.* (*Pif.*) *evansi* (n = 42). Both *Lu.* (*Lu.*) *longipalpis* and *Pi.* (*Pif.*) *evansi* have previously been reported in Honduras and implicated as vectors of *L.* (*L.*) *infantum chagasi* [7,10]. These findings are consistent with those of Raymond et al. [40], who identified *Lu*. *longipalpis* and *Pi*. *evansi* as the predominant sandfly species in the Pacific region of Nicaragua, an area that shares ecological characteristics with southern Honduras. Changes in dispersal patterns, deforestation, or shifts in sandfly population dynamics may explain the predominance of these species in central Honduras [6,39]. Although Ojos de Agua exhibited the highest Shannon diversity index (H’ = 1.2), this finding should be interpreted cautiously because of the relatively small sample size and the presence of several rare species (n ≤ 1). These factors may affect the stability and comparability of diversity estimates among study sites. In the absence of rarefaction analyses or confidence intervals, differences in diversity should not be considered conclusive evidence of ecological differences among sites. Nevertheless, the observed value may indicate a relatively heterogeneous sandfly assemblage in Ojos de Agua, although this interpretation should be confirmed through more balanced sampling.

Among the blood-engorged sandfly specimens collected, only *Lu*. *longipalpis* and *Pi*. *evansi* exhibited anthropophilic feeding behavior. This finding is consistent with previous reports from Colombia, Brazil, and Honduras [6,41,42] and further supports the potential role of these species in the transmission of NUCL and VL. Notably, a subset of these specimens tested positive for *Leishmania infantum*, further emphasizing their epidemiological relevance as potential vectors in peridomestic transmission cycles. In addition to human blood, blood meals from pigs, horses, and cattle were identified, indicating that these sandfly species feed on multiple vertebrate hosts and suggesting that local domestic animals may contribute to sustaining transmission cycles in peridomestic environments. No dog DNA was detected in any of the engorged female sand flies analyzed. This finding may reflect sampling bias, seasonal variation, or host-feeding preferences. Furthermore, many dogs in the surveyed communities sleep indoors and accompany their owners into forested areas during the day, suggesting that exposure to infected sand flies may occur outside the household environment. Therefore, the absence of canine blood meals in the analyzed specimens does not exclude the epidemiological role of dogs but may instead reflect spatial and temporal variation in vector–host interactions.

*Leishmania infantum* was the only species detected in both dogs and the sandfly vectors, *Lu. longipalpis* and *Pi. evansi*. The detection of *Leishmania infantum* DNA in multiple sandfly species and domestic reservoirs highlights the complexity of the local cycle. However, the detection of *L. infantum* DNA in sand flies alone does not demonstrate vector competence because it cannot distinguish between infective parasites and residual DNA from degraded or excreted parasites [43]. Nevertheless, these findings indicate active circulation of the parasite within the local community. Previous studies have also detected *L. infantum* DNA in *Lu. longipalpis* and *Pi. evansi* [10] and in dogs [17] from NUCL-endemic areas, suggesting that this clinical form may become established in the municipality of Ojos de Agua. However, additional surveillance studies are needed to confirm this possibility. Collectively, these findings support the potential establishment of a peridomestic transmission cycle in Ojos de Agua and underscore the need to strengthen surveillance and control activities to reduce the risk of human NUCL in the region. Beyond dogs, alternative peridomestic and sylvatic hosts may also contribute to local transmission, as documented in European foci where hares and rodents played important roles in sustaining a major urban outbreak. More broadly, the increasing frequency of zoonotic spillover at human–animal-environment interfaces, driven by expanding human populations and encroachment into wildlife habitats, underscores the importance of integrated surveillance in this setting [44,45]. As emphasized by Vincent and Gonzalez [46], monitoring parasites at these shared ecological interfaces is essential for detecting cross-species transmission before human disease becomes clinically apparent.

This study has several limitations that should be considered when interpreting the findings. First, the cross-sectional design provides only a snapshot of sandfly diversity, infection status, and canine exposure, precluding the assessment of temporal variations in transmission dynamics or causal relationships. Second, the relatively small sample size, particularly for the molecular analysis of blood-fed sand flies and the evaluation of dogs, may limit the representativeness and generalizability of the findings. Consequently, longitudinal studies incorporating larger sample sizes and broader temporal and geographic coverage are needed to better characterize *Leishmania* transmission dynamics in the region.

## 5. Conclusions

This study provides evidence suggestive of zoonotic transmission of *Leishmania infantum* in central Honduras. The identification of sandfly blood meals from humans and several domestic mammals, including pigs, cattle, and equids, suggests the existence of a potential transmission cycle involving multiple vertebrate hosts. Given this complexity, integrating a One Health approach is essential for understanding transmission dynamics, identifying the emergence of zoonotic transmission cycles, and assessing the risk of human infection. Furthermore, the detection of *L. infantum* in dogs and two anthropophilic sandfly species provides strong evidence supporting their role in the zoonotic transmission cycle of leishmaniasis.

## Figures and Tables

**Table 1:** Clinical data of households with dogs living with CL patients in Ojos de Agua, Comayagua, Honduras, 2024 (*n* = 43).

	Weight Loss	Onychogryphosis	Skin Lesion	Alopecia
Positive	30 (70.0%)	2 (4.6%)	3 (6.9%)	8 (18.6%)
Negative	13 (30.0%)	41 (95.4%)	40 (93.1%)	35 (81.4%)

**Table 2:** Serological and parasitological diagnosis of households with dogs living with CL patients in Ojos de Agua, Comayagua, Honduras, 2024 (*n* = 43).

	Immunological Diagnosis		Parasitological Diagnosis
			Peripheral Blood
	DPP	ELISA	PCR-BC
Positive	3 (7.0%)	5 (11.6%)	10 (23.2%)
Negative	40 (93.0%)	38 (88.4%)	33 (76.8%)

**Table 3:** Sandfly species collected by locality, sex, and blood meal sources.

Locality (*n*)	Species	Females	Males	Engorged Females	Total
La Ciénaga (511)	*Dampfomyia* (*Coromyia*) *zeledoni*/*deleoni*	5	2	2	7
	*Psathyromyia* (*Forattiniella*) *barrettoi majuscula*	0	1	0	1
	*Lutzomyia* (*Lutzomyia*) *longipalpis*	40	447	33	487
	*Lutzomyia* sp.	7	7	1	14
	*Pintomyia* (*Pifanomyia*) *evansi*	2	0	2	2
San Rafael (33)	*Dampfomyia* (*Coromyia*) *zeledoni*/*deleoni*	24	0	0	24
	*Lutzomyia* (*Tricholateralis*) *cruciata*	3	0	0	3
	*Psychodopygus panamensis*	1	0	0	1
	*Lutzomyia* (*Helcocyrtomyia*) *sanguinária*	1	0	0	1
	*Lutzomyia* sp.	1	0	0	1
	*Lutzomyia* (*Lutzomyia*) *longipalpis*	2	0	2	2
Ojos de Agua (101)	*Dampfomyia* (*Coromyia*) *zeledoni*/*deleoni*	23	0	1	23
	*Pintomyia* (*Pifanomyia*) *evansi*	41	0	5	41
	*Lutzomyia* (*Lutzomyia*) *longipalpis*	29	0	14	29
	*Lutzomyia* sp.	6	1	4	7
	*Micropygomyia* (*Sauromyia*) *trinidadensis*	1	0	0	1
	Total	186	458		644

**Table 4:** Blood meal sources identified in *Lutzomyia longipalpis* and *Pintomyia evansi*.

Locality (*n*)	Species	*n*	*Homo sapiens* (%)	*Equus caballus* (%)	*Sus scrofa* (%)	*Bos taurus* (%)
La Ciénaga	*Lutzomyia longipalpis*	15	9 (60.0%)	0	5 (33.3%)	1 (6.7%)
	*Pintomyia evansi*	2	2 (100%)	0	0	0
San Rafael	*Lutzomyia longipalpis*	1	1 (100%)	0	0	0
Ojos de Agua	*Pintomyia evansi*	5	5 (100%)	0	0	0
	*Lutzomyia longipalpis*	8	3 (37.5%)	1 (12.5%)	4 (50%)	0

## Data Availability

The original contributions presented in this study are included in the article. Further inquiries can be directed to the corresponding author. The newly generated sequences were submitted to the GenBank database under the accession numbers cited in the tex.

## Abbreviations

UCL	Ulcerative Cutaneous Leishmaniasis
ML	Mucosal Leishmaniasis
NUCL	Non-Ulcerated Cutaneous Leishmaniasis
VL	Visceral Leishmaniasis
DPP^®^	Dual-Path Platform
BC	Buffy Coat
